# Inherited coding variants at the *CDKN2A* locus influence susceptibility to acute lymphoblastic leukaemia in children

**DOI:** 10.1038/ncomms8553

**Published:** 2015-06-24

**Authors:** Heng Xu, Hui Zhang, Wenjian Yang, Rachita Yadav, Alanna C. Morrison, Maoxiang Qian, Meenakshi Devidas, Yu Liu, Virginia Perez-Andreu, Xujie Zhao, Julie M. Gastier-Foster, Philip J. Lupo, Geoff Neale, Elizabeth Raetz, Eric Larsen, W. Paul Bowman, William L. Carroll, Naomi Winick, Richard Williams, Torben Hansen, Jens-Christian Holm, Elaine Mardis, Robert Fulton, Ching-Hon Pui, Jinghui Zhang, Charles G. Mullighan, William E. Evans, Stephen P. Hunger, Ramneek Gupta, Kjeld Schmiegelow, Mignon L. Loh, Mary V. Relling, Jun J. Yang

**Affiliations:** 1 Department of Pharmaceutical Sciences, St. Jude Children's Research Hospital, Memphis, Tennessee 38105, USA; 2 Department of Laboratory Medicine, National Key Laboratory of Biotherapy/Collaborative Innovation Center of Biotherapy, and Cancer Center, West China Hospital, Sichuan University, Chengdu, Sichuan 610041, China; 3 Department of Pediatrics, The first affiliated hospital of Guangzhou Medical University, Guangzhou, Guangdong 510120, China; 4 Centre for Biological Sequence Analysis, The Technical University of Denmark, Kgs, Lyngby DK-2800, Denmark; 5 Department of Epidemiology, Human Genetics, and Environmental Sciences, School of Public Health, University of Texas Health Science Center, Houston, Texas 77030, USA; 6 Department of Biostatistics, Epidemiology and Health Policy Research, College of Medicine, University of Florida, Gainesville, Florida 32610, USA; 7 Department of Computational Biology, St. Jude Children's Research Hospital, Memphis, Tennessee 38105, USA; 8 Department of Pathology and Laboratory Medicine, Nationwide Children's Hospital, and Departments of Pathology and Pediatrics, Ohio State University College of Medicine, Columbus, Ohio 43205, USA; 9 Department of Pediatrics, Texas Children's Cancer Center, Baylor College of Medicine, Houston, Texas 77030, USA; 10 Hartwell Center for Bioinformatics & Biotechnology, St. Jude Children's Research Hospital, Memphis, Tennessee 38105, USA; 11 Huntsman Cancer Institute, The University of Utah, Salt Lake City, Utah 84112, USA; 12 Maine Children's Cancer Program, Scarborough, Maine 04074, USA; 13 Cook Children's Medical Center, Ft. Worth, Texas 38754, USA; 14 Pediatric Oncology, Cancer Institute New York University, New York City, New York 10016, USA; 15 Pediatric Hematology/Oncology, University of Texas Southwestern Medical Center, Dallas, Texas 75235, USA; 16 Puma Biotechnology Inc., Los Angeles, California 90024, USA; 17 The Novo Nordisk Foundation Center for Basic Metabolic Research, Faculty of Health and Medical Sciences, University of Copenhagen, Copenhagen DK-2200, Denmark; 18 Department of Pediatrics, The Children's Obesity Clinic, Copenhagen University Hospital Holbaek, Holbaek DK-4300, Denmark; 19 McDonnell Genome Institute, Washington University School of Medicine, St Louis, Missouri 63108, USA; 20 Hematological Malignancies Program, Comprehensive Cancer Center, St. Jude Children's Research Hospital, Memphis, Tennessee 38105, USA; 21 Department of Oncology, St. Jude Children's Research Hospital, Memphis, Tennessee 38105, USA; 22 Department of Pathology, St. Jude Children's Research Hospital, Memphis, Tennessee 38105, USA; 23 Division of Oncology and Center for Childhood Cancer Research, Children's Hospital of Philadelphia, Philadelphia, Pennsylvania 19104, USA; 24 Department of Paediatrics and Adolescent Medicine, The Juliane Marie Centre, The University Hospital Rigshospitalet, and the Institute of Clinical Medicine, Faculty of Health, University of Copenhagen, Copenhagen DK-2100, Denmark; 25 Department of Pediatrics, Benioff Children's Hospital and the Helen Diller Family Comprehensive Cancer Center, University of California at San Francisco, San Francisco, California 94115, USA

## Abstract

There is increasing evidence from genome-wide association studies for a strong inherited genetic basis of susceptibility to acute lymphoblastic leukaemia (ALL) in children, yet the effects of protein-coding variants on ALL risk have not been systematically evaluated. Here we show a missense variant in *CDKN2A* associated with the development of ALL at genome-wide significance (rs3731249, *P*=9.4 × 10^−23^, odds ratio=2.23). Functional studies indicate that this hypomorphic variant results in reduced tumour suppressor function of p16^INK4A^, increases the susceptibility to leukaemic transformation of haematopoietic progenitor cells, and is preferentially retained in ALL tumour cells. Resequencing the *CDKN2A*–*CDKN2B* locus in 2,407 childhood ALL cases reveals 19 additional putative functional germline variants. These results provide direct functional evidence for the influence of inherited genetic variation on ALL risk, highlighting the important and complex roles of *CDKN2A*–*CDKN2B* tumour suppressors in leukaemogenesis.

## Abstract (web summary)

Genome-wide association studies indicate a strong genetic susceptibility to acute lymphoblastic leukaemia in children, though the effect on protein-coding genes is not fully understood. Here Xu and Zhang *et al.* identify a missense variant in *CDKN2A* which reduces tumour suppression.

## Graphical abstract

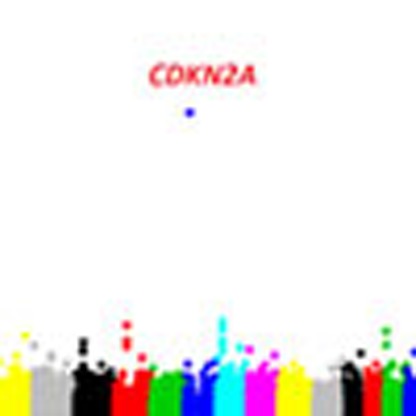

The risk of developing acute lymphoblastic leukaemia (ALL) is highest between 2 and 5 years after birth^1^,^2^, with initiating sentinel somatic genomic lesions (for example, chromosomal translocations) detectable at the time of birth in many cases^3^,^4^. This early disease onset suggests a strong inherited genetic basis for ALL susceptibility, and recent genome-wide association studies (GWAS) have discovered at least six risk loci: *ARID5B*, *IKZF1*, *CEBPE*, *PIP4K2A-BMI1*, *GATA3* and *CDKN2A*–*CDKN2B*^5^,^6^,^7^,^8^,^9^,^10^. These ALL risk genes are directly involved in haematopoietic stem cell function, lymphocyte differentiation and development, and cell cycle regulation^11^,^12^,^13^,^14^,^15^, several of which are also commonly targeted by somatic genomic lesions. In particular, the *CDKN2A*–*CDKN2B* locus is one of the most frequently deleted genomic regions in childhood ALL with focal copy number loss in both B- and T-cell ALL^14^,^16^.

The vast majority of variants examined in previous ALL GWAS are intronic or intergenic. Although it is now evident that non-coding variants related to disease traits are significantly over-represented in regulatory DNA and often function as modulators of local or distal gene transcription^17^,^18^, questions also arise whether coding variants within ALL susceptibility genes might confer even greater effects on disease development. Moreover, a large number of low-frequency and rare-coding germline variants have been discovered by exome-sequencing efforts^19^, but their contributions to ALL pathogenesis have yet to be examined systematically.

In the present study, we perform an exome-focused GWAS to systematically examine the impact of germline-coding variants on the development of ALL in children of European descent, experimentally explore the functional consequences of the genome-wide significant variant in the *CDKN2A* gene, and comprehensively characterize coding variation at this locus by targeted resequencing.

## Results

### Exome-focused GWAS of ALL susceptibility

**Figure 1:**
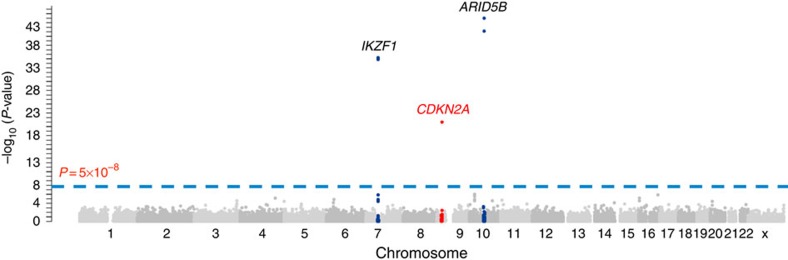
GWAS results of ALL susceptibility in European Americans. Association between genotype and ALL was evaluated for 35,802 SNPs in 1,773 ALL cases and 10,448 non-ALL controls. *P*-values (the logistic regression test, −log_10_ *P*, *y* axis) were plotted against respective chromosomal position of each SNP (*x* axis). Gene, symbols were indicated for 3 loci achieving genome-wide significance threshold (*P*<5 × 10^−8^, dashed blue line): *ARID5B* (10q21.2), *IKZF1* (7p12.2) and *CDKN2A* (9p21.3). Blue dots indicated SNPs within 2M bp of the top ALL susceptibility variants at the *ARID5B* (rs10821936) and *IKZF1*(rs4132601) loci, the red dots indicated SNPs in the 2M-bp region around the novel ALL risk variant rs3731249 in *CDKN2A*.

In the discovery GWAS, we genotyped 1,773 children with B-ALL and 10,448 non-ALL controls of European descent^20^,^21^ for 247,505 variants using the Illumina Infinium HumanExome array. Three loci with genome-wide significant association signals were observed: *ARID5B* (10q21.2), *IKZF1* (7q12.2) and *CDKN2A* (9p21.3) (Fig. 1). Non-coding variants rs10821936 in *ARID5B* and rs4132601 in *IKZF1* showed the strongest association (*P*=9.9 × 10^−46^ and 4.3 × 10^−37^, the logistic regression test, respectively; Fig. 1 and Supplementary Table 1), confirming previous GWAS findings from our group and others^5^,^6^. No coding variants in *ARID5B* and *IKZF1* were significantly associated with ALL susceptibility. The third genome-wide significant hit was a missense SNP at the *CDKN2A* locus (rs3731249, *P*=9.4 × 10^−23^, the logistic regression test, Fig. 1, Table 1). The T allele at rs3731249 was over-represented in ALL compared with controls (6.8% versus 3.0%, Table 1), with every copy of the allele conferring 2.23-fold increase in disease risk (95% confidence interval 1.90–2.61). The C-to-T nucleotide substitution at rs3731249 (c.C442T) resulted in an alanine-to-threonine change in amino-acid sequence (p.A148T) for tumour suppressor p16^INK4A^. This variant also locates in the 3′ untranslated region (3′-UTR) of the p14^ARF^ transcript, an alternative open reading frame at this locus encoding a different tumour suppressor. Interestingly, previous GWAS had identified an intronic variant in *CDKN2A* (rs3731217) to be strongly associated with susceptibility to ALL in populations of European descent^9^. Genotype correlation between the coding variant rs3731249 and the intronic rs3731217 is exceedingly low (*r*^2^<0.01 in Europeans, Supplementary Fig. 1), and multivariate analyses including both SNPs indicated their independent contribution to ALL risk (Supplementary Table 2). In the replication cohort of 409 childhood ALL cases and 1,599 non-ALL controls of European descent in Denmark, the association signal at rs3731249 was validated (*P*=5.2 × 10^−4^, odds ratio=1.73 (1.27–2.36), the logistic regression test, Table 1) and this variant also remained significant after adjusting for rs3731217.

### Functional characterization of the rs3731249 variant

**Figure 2:**
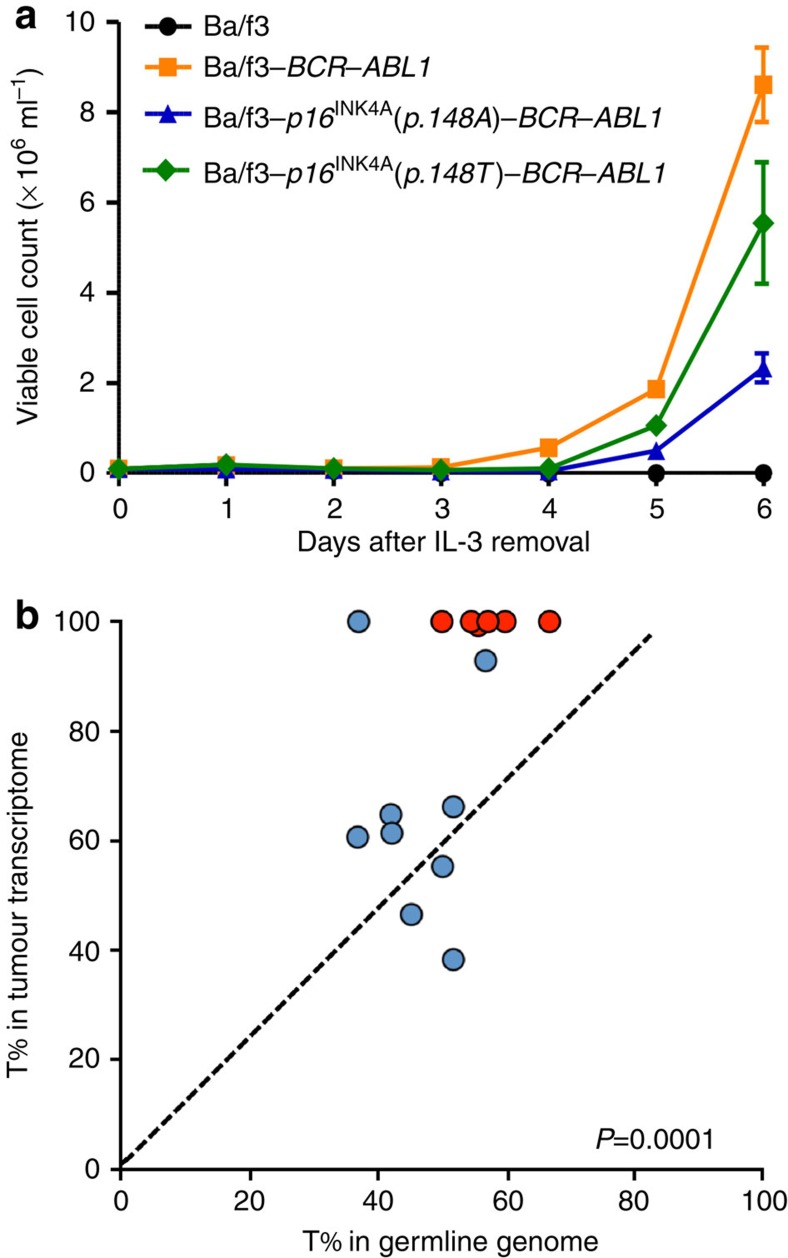
Functional characterization of ALL risk variant at rs3731249. (**a**) Mouse haematopoietic progenitor cell Ba/f3 overexpressing wildtype, variant p16^INK4A,^ or transfected with control vector was transduced with leukaemia oncogenic *BCR*–*ABL1* fusion gene. Cell proliferation in the absence of cytokine IL3 was measured daily as an indicator of leukaemic transformation. Ectopic expression of p16^INK4A^ (p.148 T, green) significantly potentiated leukaemic transformation by *BCR*–*ABL1,* compared with cells expressing wild-type p16^INK4A^ (p.148A, blue), consistent with the association of this allele with susceptibility to ALL. Data represent the mean of three replicates±s.e.m. **(b)** Allele-specific expression of p16^INK4A^ in ALL blasts was determined by comparing the number of sequence reads for transcripts containing C or T alleles at rs3731249 (*p16*^*INK4A*^p.148A versus *p16*^*INK4A*^p.148 T), in 15 childhood ALL cases with heterozygous genotype in the germline DNA at this SNP. Each dot represents an ALL case (red indicates cases with somatic deletion (loss of heterozygosity) and blue indicates cases without copy number change in tumour) and the line of identity indicates equal expression of both alleles. *P*-value was estimated by paired *t*-test based on the number of sequence reads for each allele.

To experimentally evaluate the effects of rs3731249 on ALL leukaemogenesis, we directly compared the effect of wildtype versus variant allele p16^INK4A^ (p.148A versus p.148 T) on *BCR*–*ABL1*-mediated leukaemic transformation *in vitro*. We chose mouse haematopoietic progenitor Ba/f3 cell line because it is inherently p16^Ink4a^-defective due to methylation at the *Ink4a-Arf* locus^22^, and ectopic expression of *BCR-ABL1* in Ba/f3 cells efficiently induces exogenous cytokine (interleukin 3 (IL3))-independent proliferation. Over-expression of wild-type p16^INK4A^(p.148A) significantly inhibited leukaemic transformation by *BCR*–*ABL1* (Fig. 2a, Supplementary Fig. 2), consistent with its role as a critical tumour suppressor in ALL. In contrast, Ba/f3 cells overexpressing variant p16^INK4A^(p.148 T) were significantly more susceptible to *BCR*–*ABL1* transformation measured by IL3-independent growth, suggesting that the p.148 T variant is likely hypomorphic with reduced tumour suppressor function. In Ba/f3 cells transfected with both variant and wild-type *p16*^*INK4A*^, the relative ratio of the p.148 T (variant) to p.148A (wildtype) transcript increased substantially upon *BCR*–*ABL1*-mediated transformation (Supplementary Fig. 3), consistent with the increased leukaemia risk conferred by the variant allele at rs3731249. To further examine the potential susceptibility to ALL conferred by the rs3731249 in patients, we compared the genotype distribution in RNA and DNA from primary leukaemic blasts and matched germline samples from children with ALL (Fig. 2b). Of 15 cases with the heterozygous germline genotype at this SNP, six exhibited somatic deletion of one copy of *CDKN2A*, all of which retained the risk allele in tumour cells. Even in cases not affected by somatic copy number loss at this locus, the variant *p16*^*INK4A*^(c.442 T) was preferentially transcribed relative to wildtype (c.442C), with allele-biased expression ranging from 61 to 100%, Fig. 2b). Altogether, these results pointed to the possibility that cells carrying the hypomorphic risk allele at rs3731249 might have been enriched during leukaemogenesis.

### Targeted resequencing of *CDKN2A* and *CDKN2B* in childhood ALL

**Figure 3:**
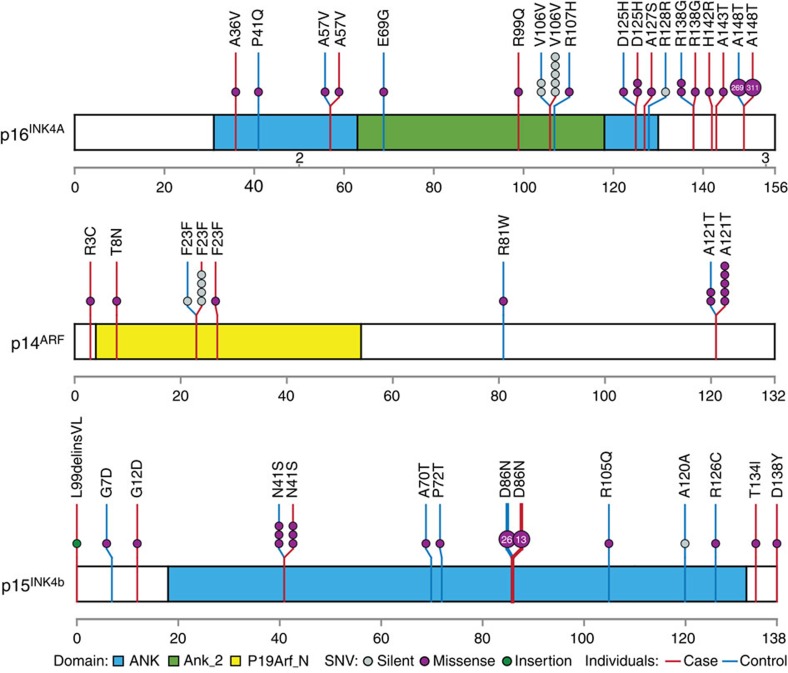
Targeted resequencing of *CDKN2A–CDKN2B* locus identified additional germline coding variants in children with ALL. *CDKN2A* and *CDKN2B* genes were sequenced using Illumina HiSeq platform following capture-based enrichment of this genomic region in 2,407 ALL cases of European descent. Variants in non-ALL controls were based on publicly available data from the individuals of European descent within the NHLBI Exome Sequencing Project (*N*=4,300). Exonic variants are classified as silent or missense (grey or purple solid circles) and are mapped to three distinct open reading frames at this locus: p16^INK4A^, p14^ARF^ and p15^INK4B^, for ALL cases (red vertical lines) and non-ALL controls (blue vertical lines), and functional domains are indicated by colour based on Pfam annotation. Each circle represents a unique individual carrying the indicated variant (heterozygous or homozygous), except for variants recurring in more than 10 individuals for which the number in the circle indicates the exact frequency of the observed variant.

To comprehensively identify putative functional ALL susceptibility variants at this locus, we resequenced the coding region of the *CDKN2A* and *CDKN2B* genes in germline DNA from 2,407 childhood ALL cases (1,450 of which were also included in the discovery GWAS). In addition to rs3731249, we observed another 13 germline exonic variants in tumour suppressors p16^INK4A^ and p14^ARF^ encoded by the *CDKN2A* gene, 12 of which result in amino-acid sequence changes (Fig. 3, Supplementary Table 3). These missense variants were all singletons, except for the p.D125H variant in p16^INK4A^ and the p.A121T variant in p14^ARF^ observed in two and five cases, respectively. Five variants were predicted to be damaging based on combined annotation dependent depletion^23^ (CADD score>13, Supplementary Table 3), and we did not observe germline insertions or deletions in *CDKN2A* in our ALL cohort. Comparing with 4,300 European American individuals from the NHLBI GO Exome Sequencing Project (ESP), there was a trend for a higher burden of rare missense variants in relative to controls the *CDKN2A* gene (p16^INK4A^ and p14^ARF^) in children with ALL (0.71% versus 0.23%, *P*=0.0045, Fisher's exact test, Fig. 3). In addition, we identified six germline-coding variants in the adjacent *CDKN2B* gene in this cohort of children with ALL, although there was no significant over-representation compared with European controls in the ESP cohort (0.83% versus 0.79%, Fig. 3).

## Discussion

Encoding three tumour suppressor proteins (p16^INK4A^, p14^ARF^ and p15^INK4B^), the *CDKN2A*–*CDKN2B* locus at 9p21 is promiscuously associated with tumorigenesis and commonly targeted by somatic mutation, deletion and/or hypermethylation in various cancers. p16^INK4A^ and p15^INK4B^ are highly homologous inhibitors of cyclin-dependent kinase and function mainly as master regulators of cell cycle entry via the Rb-E2F signalling axis^24^. Although also encoded by the *CDKN2A* gene, p14^ARF^ utilizes a completely different reading frame with distinct tumour suppression functions by inhibiting MDM2 and activating p53^25^. Suppressed during normal haematopoiesis, p16^INK4A^ and p14^ARF^ expression is activated on oncogenic stimuli (for example, constitutive expression of *BCR-ABL1* fusion) to trigger cell cycle exit (senescence) or apoptosis as a means of eliminating oncogene-stressed cells^26^. In fact, the *CDKN2A*–*CDKN2B* locus is either bi- or monoallelicly deleted in 64% of *BCR*–*ABL1*-positive ALL cases and in 32–72% of T- or B-ALL cases without the *BCR-ABL1* translocation, suggesting positive selection for cells with defective p16^INK4A^, p14^ARF^ and p15^INK4B^ (or some combinations thereof) during leukaemogenesis.

The previously reported ALL susceptibility variant rs3731217 is located in a non-coding region downstream of exon 1β (specific for p14^ARF^), but distal to exon 1α (specific for p16^INK4A^) of the *CDKN2A* gene. The germline genotype at this SNP was not associated with overall *CDKN2A* expression in lymphoblastoid cell lines^9^ but transcript-specific analyses may be needed to definitively determine the effects of this variant on p14^ARF^ versus p16^INK4A^ expression. In contrast, the genome-wide significant variant rs3731249 in our current GWAS localizes to exon 2 of *CDKN2A*. While this exon is shared by both p16^INK4A^ and p14^ARF^, the C-to-T nucleotide transition causes a missense change for the p16^INK4A^ open reading frame but is in the UTR of the p14^ARF^, therefore, likely to have a more direct effect on the former. This hypothesis is supported by the fact that haematopoietic progenitor cells (Ba/f3) expressing variant p16^INK4A^ were substantially more susceptible to *BCR*–*ABL1*-mediated leukaemic transformation compared with cells with the wild-type protein (Fig. 2a), pointing to rs3731249 as a possible functional variant directly contributing to the association with ALL risk. The structural basis of the hypomorphic effects of the p.A148T variant is unclear, since this residue is not directly involved in binding to CDK4 or CDK6^27^. However, there was evidence that the variant p16^INK4A^ (p.148 T) is preferentially retained in the nucleus compared with the wild-type p16^INK4A^ (p.148A), compromising its ability to inhibit CDKs in the cytoplasm^28^,^29^. The relative contribution of p16^INK4A^ versus p14^ARF^ to ALL pathogenesis is not unequivocal because somatic deletions at this locus almost always lead to the loss of both genes. Although the rs3731249 variant also results in sequence changes of the 3′-UTR of the *p14*^*ARF*^ transcript, bioinformatic prediction did not identify any potential effects on mRNA stability or microRNA binding and no difference was observed in reporter gene transcription under the influence of 3′-UTR containing either the wildtype or variant allele at rs3731249 (Supplementary Fig. 4), suggesting minimal effects of this variant on *p14*^*ARF*^ transcription. Finally, rs3731249 is also observed in non-European populations, for example, there was a trend for a higher frequency of the risk allele in African American children with ALL than that in individuals from this racial background in the NHLBI ESP cohort (0.58% in 260 ALL cases versus 0.38% in 2,203 controls), although a much larger sample size is needed to rigorously examine the statistical significance of such differences. It should be noted that we and others previously showed that the non-coding ALL risk variants (rs17756311 and rs3731217) at this locus had much stronger effects in European Americans than in other race/ethnic groups^7^,^30^, suggesting potential racial differences in genetic susceptibility to ALL.

We subsequently identified additional coding variants in p16^INK4A^, p14^ARF^ and p15^INK4B^ by resequencing, most of which were low frequency or rare. While there was a modest over-representation of potentially damaging coding variants in ALL cases compared with controls (Fig. 3), our data do not suggest that rare variants contribute substantially to the associations with ALL susceptibility observed at this locus. It should also be noted that the vast majority of coding variants within the *CDKN2A* gene affects only one of the two tumour suppressors (either p16^INK4A^ or p14^ARF^). Interestingly, rs199888003 is the only variant that is located in the coding region of both p16^INK4A^ and p14^ARF^, resulting in an alanine-to-threonine change in p14^ARF^ (p.A121T) with synonymous effect on p16^INK4A^. This is also the most frequent germline missense variant in p14^ARF^ in our cohort and was over-represented in ALL compared with non-ALL controls (0.21% versus 0.046%, respectively, Fig. 3). This substitution of threonine in p14^ARF^ adds a possible glycosylation and phosphorylation site and also introduces a phosphoprotein-binding FHA domain implicated in DNA damage response and cell cycling^31^. Future studies are warranted to determine the exact consequences of this variant on p14^ARF^ functions. To systemically evaluate the contribution of low frequency and rare-coding variants to ALL risk, we also performed genome-wide gene-level burden test but did not observe any genome-wide significant associations (Supplementary Table 4). Of the six known ALL risk loci, we noted two coding variants in *CEBPE* (rs141903485 and rs146580935, Supplementary Table 5) nominally associated with ALL susceptibility.

In conclusion, we comprehensively evaluated exonic genetic variations for association with ALL susceptibility and identified novel coding risk variants at the *CDKN2A*–*CDKN2B* locus that may directly affect tumour suppressor functions and potentiate leukaemic transformation. These results provided functional evidence for the influence of inherited genetic variants on ALL leukaemogenesis, further indicating that a continuum of genetic variations in both host and tumour genomes contribute to malignant transformation and cancer risk.

## Methods

### Subjects and samples

The discovery GWAS consisted of 1,773 childhood B-ALL cases and 10,448 non-ALL controls of European descent (>90% European genetic ancestry as estimated using STRUCTURE^32^,^33^). ALL cases were from the Children's Oncology Group (COG) AALL0232 study (*N*=1,277)^8^, the COG P9906 protocol (*N*=115)^34^ and St Jude Total Therapy XIIIB and XV protocols (*N*=381)^5^. Unrelated individuals of European descent from the Atherosclerosis Risk in Communities (ARIC) study^20^,^21^ were used as non-ALL controls because the prevalence of adult survivors of childhood ALL is less than 1 in 10,000 in the US. The replication series included 409 children with ALL from NOPHO ALL92, ALL2000 and ALL2008 protocols^35^ and 1,599 unrelated non-ALL controls from Danish Childhood Obesity Biobank study (clinicaltrials.gov: NCT00928473) in Holbæk and at random schools in Zealand, Denmark. ALL cases were selected only on the basis of sample availability, and we did not observe any statistically significant differences in demographic or clinical features of children included versus not included in this genetic study. We elected to focus on individuals of European descent to minimize population stratification^36^.

Germline DNA for cases was extracted from peripheral blood or bone marrow samples obtained during clinical remission (<5% ALL blasts by morphology). This study was approved by the Institutional Review Board at St Jude Children's Research Hospital and COG member institutions and the Ethics Committee at the Danish Data Protection Agency, Region Zealand and the University Hospital Rigshospitalet, Denmark. Informed consent was obtained from parents, guardians, or patients, as appropriate.

### Genotyping and quality control

SNP genotyping was performed in germline DNA using the Illumina Infinium HumanExome Array v1.0 in the discovery GWAS, and using Illumina HumanCoreExome chip for the replication series. Genotype calls (coded as 0, 1, and 2 for AA, AB and BB genotypes) were determined using the Illumina GenomeStudio Software. For the ALL cases, samples for which genotype was ascertained at <98% of SNPs on the array were deemed to have failed and were excluded from the analyses. Quality control procedures were performed for both samples and SNPs on the basis of call rate, minor allele frequency (MAF), and Hardy Weinberg equilibrium (Supplementary Fig. 5). Detailed quality control for the non-ALL controls from the ARIC study was performed at the University of Texas Health Science Center following established protocols^21^.

We performed principal component analysis of cases and controls in the discovery GWAS to characterize population substructure (Supplementary Fig. 6).

### Genome-wide analyses

In the discovery GWAS, the association of each SNP individually with ALL susceptibility was tested by comparing the genotype frequency between ALL cases and non-ALL controls in logistic regression models, after adjusting for top 10 principal components to control for population stratification. A quantile–quantile (Q–Q) plot was constructed and there was only minimal inflation at the upper tail of the distribution (*λ*=1.08, Supplementary Fig. 7). In the replication studies, we evaluated the novel genome-wide significant variant rs3731249, using the same logistic regression models. Multivariate logistic regression model including both rs3731217 and rs3731249 were tested to determine independent association signals at the *CDKN2A* locus in both discovery and replication series.

We also performed gene-level analyses to evaluate the aggregated effects of low-frequency variants on ALL susceptibility, using the SKAT test^37^. Missense, stop codon-altering and splice-site variants with MAF<5% were included. In total, 12,687 genes with at least two variants were tested.

R (version 3.0) statistical software was used for all analyses unless indicated otherwise.

### *CDKN2A*–*CDKN2B* resequencing and rare variant analyses

Germline DNA from 2,407 children with ALL was used to create individual Illumina dual-indexed libraries. These libraries were pooled in sets of 96 and hybridized with a custom version of the Roche NimbleGen SeqCap EZ custom probes to capture the *CDKN2A*–*CDKN2B* region on 9p21. Quantitative PCR was used to determine the appropriate capture product titre necessary to efficiently populate an Illumina HiSeq 2000 flowcell for paired-end 2 × 101 bp sequencing. Each sequence pool of 96 samples was demultiplexed, with coverages of >20 × depth across >90% of the targeted regions for nearly all samples. Sequence reads in FASTQ format were mapped and aligned using the Burrows–Wheeler Aligner, and genetic variants were called using the GATK pipeline version 3.1 (ref. 38). We compared the proportion of rare variant-carriers in ALL subjects (either homozygous or heterozygous) versus that in individuals of European descent in the ESP cohort (non-ALL controls), focusing on variants with MAF<1%. Statistical significance of the difference was estimated using Fisher's exact test.

*CDKN2A* sequencing was also performed in matched germline and diagnostic ALL tumour DNA by Complete Genomics for all cases with available materials, and in tumour RNA by RNA-seq. Details regarding sequencing, data analysis and coverage are available at ftp://caftpd.nci.nih.gov/pub/dcc_target/ALL/Phase_II/sequence/WGS/CGI_TARGET_Pipeline_README.pdf, or as previously described^39^ (European Genome Phenome archive: EGAS00001000654).

### Leukaemic transformation assay in Ba/f3 cells

The full-length *CDKN2A* was purchased from GE Healthcare. The p.A148T variant (rs3731249) was introduced by site-directed mutagenesis (forward primer: 5′-TGCCCGCATAGATGCCACGGAAGGTCCCTCAGA-3′, reverse primer: 5′-TCTGAGGGACCTTCCGTGGCATCTATGCGGGCA-3′) and cloned into the cL20c-IRES–GFP lentiviral vector, and lentiviral supernatants containing cL20c–p16^INK4A^p.148A–IRES–GFP or cL20c–p16^INK4A^p.148 T–IRES–GFP were produced by transient transfection of 293 T cells (American Type Culture Collection) using calcium phosphate. The MSCV (Babe MCS)–*BCR*–*ABL1*–Luc2 construct was a gift from Dr Charles Sherr at St Jude Children's Research Hospital^22^ and retroviral particles were produced using 293 T cells. Ba/f3 cells (gift from Dr Omar Abdel-Wahab at the Memorial Sloan Kettering Cancer Center) were maintained in medium supplemented with 10 ng ml^−1^ recombinant mouse IL3. Ba/f3 cells were transduced with lentiviral supernatants with wild-type or variant p16^INK4A^(Supplementary Fig. 8). GFP-positive cells were sorted 48 h after transduction and maintained in IL3 medium for another 24 h before transfected by *BCR*–*ABL1* retroviral supernatants. Forty-eight hours later, cells were washed three times and grown in the absence of cytokine. Cell growth and viability were monitored daily by Trypan blue using a TC10 automated cell counter (BIO-RAD). Each experiment was performed three times.

For immunoblotting assays, Ba/f3 cells were washed and resuspended in lysis buffer (10 × PBS with 0.5 M EDTA, 10% NP-40 and 50% glycerol) with protease inhibitors and phosphatase inhibitors. Lysates were sonicated six times and centrifuged at 13,000 g for 10 min at 4 °C. Supernatants were quantified for protein concentration by BCA kit, electrophoresed, and transferred to nitrocellulose membranes. Membranes were probed with 1: 1,000 anti-p16^INK4A^ antibody (Abcam, ab81278), with α-tubulin as a loading control (1: 1,000 anti-tubulin antibody, Sigma-Aldrich, T5618).

For quantitative reverse transcription-PCR (qRT–PCR), total RNA was extracted using the RNeasy Micro kit (Qiagen) according to the manufacturer's protocol. Total RNA (500 ng) was reverse transcribed into cDNA using oligoT primers and the SuperScript III reverse transcriptase kit (Invitrogen). Quantitative real-time PCR was performed by using ABI Prism 7900HT detection system (Applied Biosystems) with Faststart SYBR Green master mix (Roche). Relative expression was calculated as a ratio of *BCR-ABL1* to *Hprt.* Primer sequences of *BCR*–*ABL1* and *Hprt* were as follows: *BCR-ABL1* (forward: 5′-CTGGCCCAACGATGGCGA-3′; reverse: 5′-CACTCAGACCCTGAGGCTCAA-3′); *Hprt* (forward: 5′-GAGCAATGATCTTGATCTTC-3′; reverse: 5′-TTCCTTCTTGGGTATGGAAT-3′).

To co-express rs3731249 variant and wild-type p16^INK4A^, Ba/f3 cells were transduced with equal molar cL20c–p16^INK4A^p.148A–IRES–GFP and cL20c–p16^INK4A^p.148 T–IRES–iYFP lentivirus and cells successfully transfected with both were selected by flow cytometry sorting for GFP/YFP double positivity. *BCR-ABL1*-mediated transformation was performed as described above. Genomic DNA and RNA samples were collected at day 0, 2, 4 and 5 after IL3 removal. p.148A and p.148 T transcript in RNA was quantified using allele-specific Taqman genotyping assay and normalized to allele ratio in matched DNA samples at respective time points. Each experiment was performed three times and each sample was assayed in triplicate.

### Luciferase reporter assays

The *p14*^*INK4A*^–3′-UTR vector (3′-UTR for Human NM_058195.2 was placed downstream of luciferase reporter gene on the pEZX-MT01 backbone) was purchased from GeneCopoeia and the T variant at rs3731249 was introduced by site-directed mutagenesis (forward primer: 5′-CCATGCCCGCATAGATGCCGTGGAAGGTCCCTCAGACATCC-3′; reverse primer: 5′-GGATGTCTGAGGGACCTTCCACGGCATCTATGCGGGCATGG-3′). For reporter gene assay, 2.5 × 10^4^ 293 T cells cultured in 96-well plate were transiently transfected with 100 ng empty vector, variant, or wild-type *p14*^*INK4A*^ 3′UTR constructs using Lipofectamine 2000 (Invitrogen). Firefly luciferase activities were measured 24 h later using the Dual Luciferase Assay (Promega). The results were normalized against Renilla luciferase. Each reporter construct transfection was replicated at least three times, and each sample was assayed in triplicate.

## Additional information

**Accession codes.** The RNA-seq data have been deposited in European Genome Phenome archive under the accession codes EGAS00001000654.

**How to cite this article:** Xu, H. *et al.* Inherited coding variants at the CDKN2A locus influence susceptibility to acute lymphoblastic leukaemia in children. *Nat. Commun.* 6:7553 doi: 10.1038/ncomms8553 (2015).

## Supplementary Material

Supplementary Information Supplementary Figures 1-8 and Supplementary Tables 1-5

## Figures and Tables

**Table 1:**
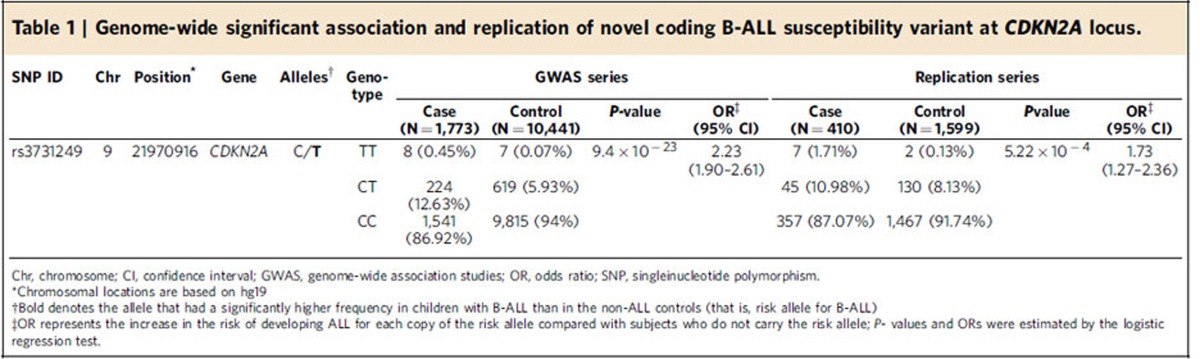
Genome-wide significant association and replication of novel coding B-ALL susceptibility variant at *CDKN2A* locus.

Chr, chromosome; CI, confidence interval; GWAS, genome-wide association studies; OR, odds ratio; SNP, singleinucleotide polymorphism.

^*^Chromosomal locations are based on hg19

^†^Bold denotes the allele that had a significantly higher frequency in children with B-ALL than in the non-ALL controls (that is, risk allele for B-ALL)

^‡^OR represents the increase in the risk of developing ALL for each copy of the risk allele compared with subjects who do not carry the risk allele; *P-* values and ORs were estimated by the logistic regression test.
