# Supplementary material for: Inherited coding variants at the CDKN2A locus influence susceptibility to acute lymphoblastic leukaemia in children
Source: Nat Commun. 2015 Jun 24;6:7553. doi: 10.1038/ncomms8553 (PMC4544058; doi:10.1038/ncomms8553)
Supplement: Supplementary Information — Supplementary Figures 1-8 and Supplementary Tables 1-5 [file ncomms8553-s1.pdf]

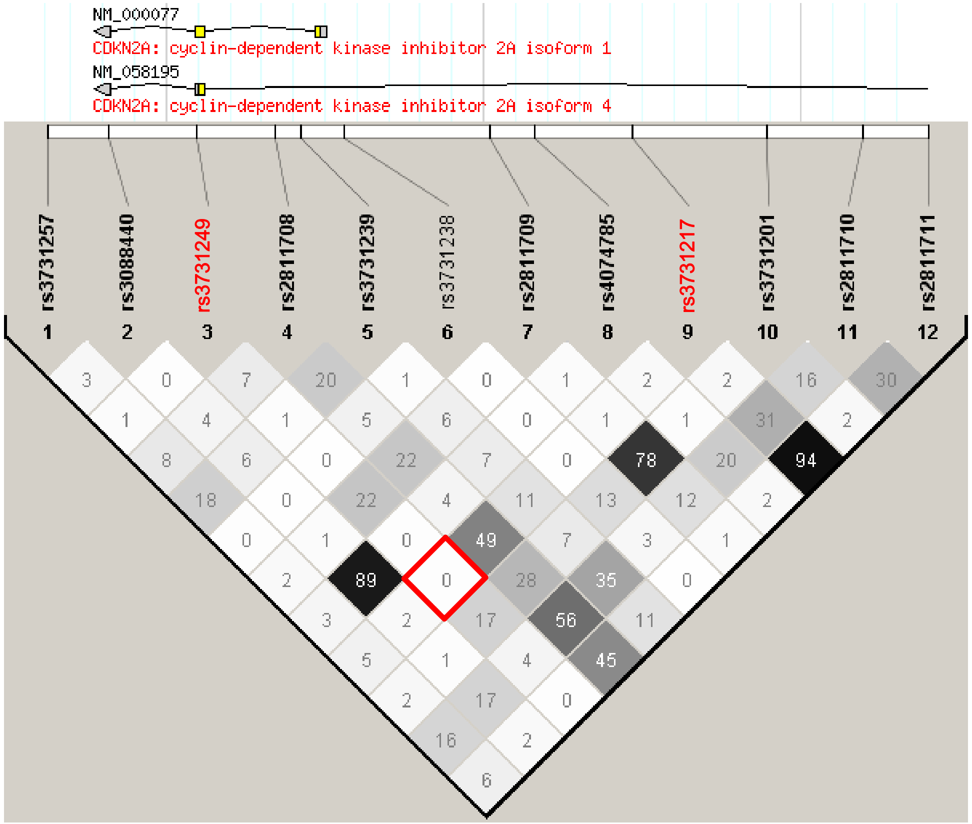


**Supplementary Figure 1. Linkage disequilibrium (LD) at the *CDKN2A* locus**. Minimal correlation was observed ($r^2$=0.0007) in Hapmap CEU individuals between B-ALL risk variants rs3731249 and the rs3731217 (highlighted in red).

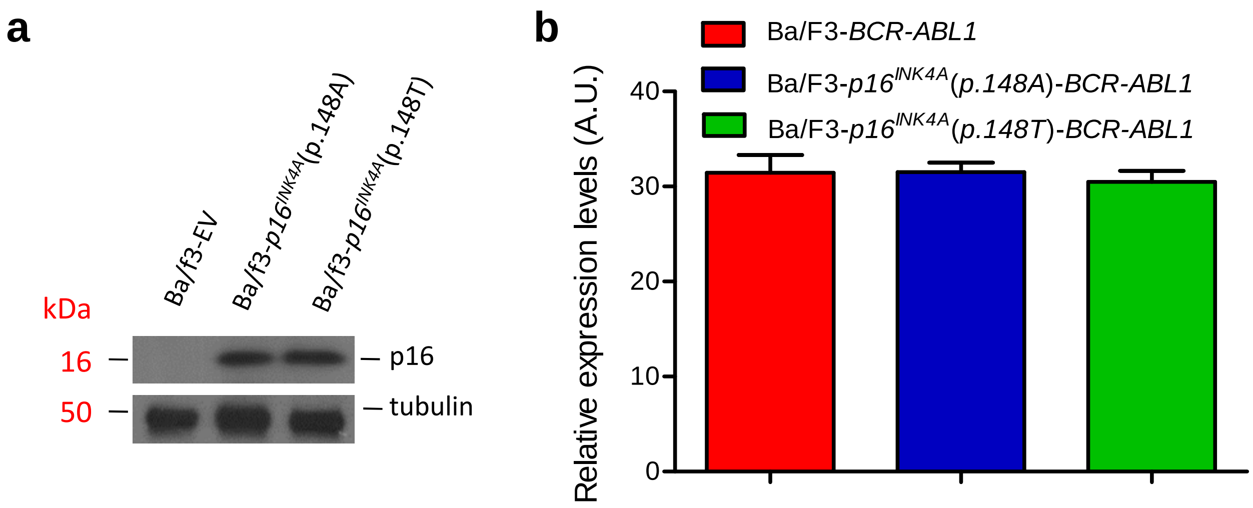


**Supplementary Figure 2. Transduction efficiency of *p16*$^{INK4A}$ and *BCR-ABL* in Ba/F3 cells.** (**a**) Western blot showed similar levels of wildtype and variant p16$^{INK4A}$ protein (p.148A vs. p.148T). α-tubulin was used as loading control. Results were confirmed by three independent experiments. (**b**) Relative *BCR-ABL1* expression level was determined in *BCR-ABL1* transformed Ba/F3 cells by real-time PCR. Data represent the mean of three replicates ± standard error of the mean (SEM).

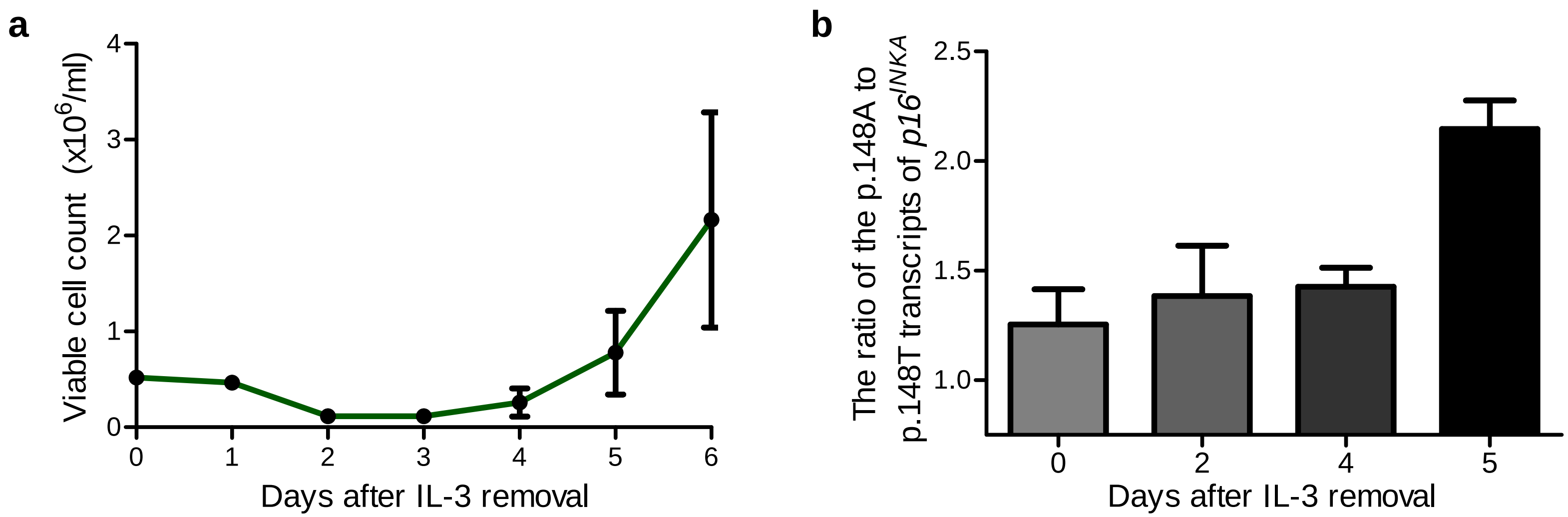


**Supplementary Figure 3. Differential expression of the wildtype (*p.148A)* vs. variant (*p.148T) p16$^{INK4A}$* transcript during *BCR-ABL1*-mediated transformation.** (**a**) *BCR-ABL1* mediated-transformation of mouse hematopoietic progenitor cell Ba/f3 co-transduced with equal molar wildtype and variant *p16$^{INK4A}$.* Ba/f3 cells were transduced with equal molar cL20c-p16$^{INK4A}$p.148A-IRES-GFP and cL20c-p16$^{INK4A}$p.148T-IRES-iYFP lentivirus and cells successfully transfected with both were selected by flow cytometry sorting for GFP/YFP double positivity. Following *BCR-ABL1* transduction, IL-3 independent growth was monitored daily until overt transformation. (**b**) Relative expression of wildtype and variant *p16$^{INK4A}$* transcript during *BCR-ABL1* mediated-transformation. Genomic DNA and RNA samples were collected at day 0, 2, 4, and 5 after IL-3 removal. p.148A and p.148T transcript was quantified using allele-specific Taqman genotyping assay and normalized to allele ratio in matched DNA samples at respective time points. Each bar represents the ratio of variant over wildtype *p16$^{INK4A}$* transcript, and levels above 1 indicate higher proportion of the variant *p16$^{INK4A}$* transcript. Data represent the mean of three replicates ± SEM.

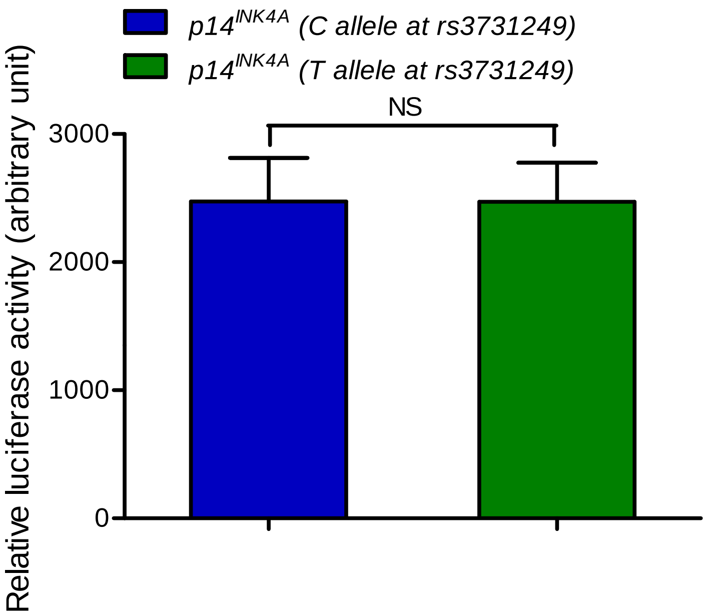


**Supplementary Figure 4**: **Effects of the rs3731249 variant on function of the *p14$^{INK4A}$ 3'UTR*.** Variant and wildtype p14$^{INK4A}$ 3'UTR was cloned downstream of luciferase reporter gene in the pEZX-MT01 backbone (GeneCopoeia). Human 293T cells were transiently transduced with wildtype or variant constructs and relative firefly luciferase units was normalized to the renilla luciferase intensity 24 hours later . Data represent the mean of three replicates ± SEM.

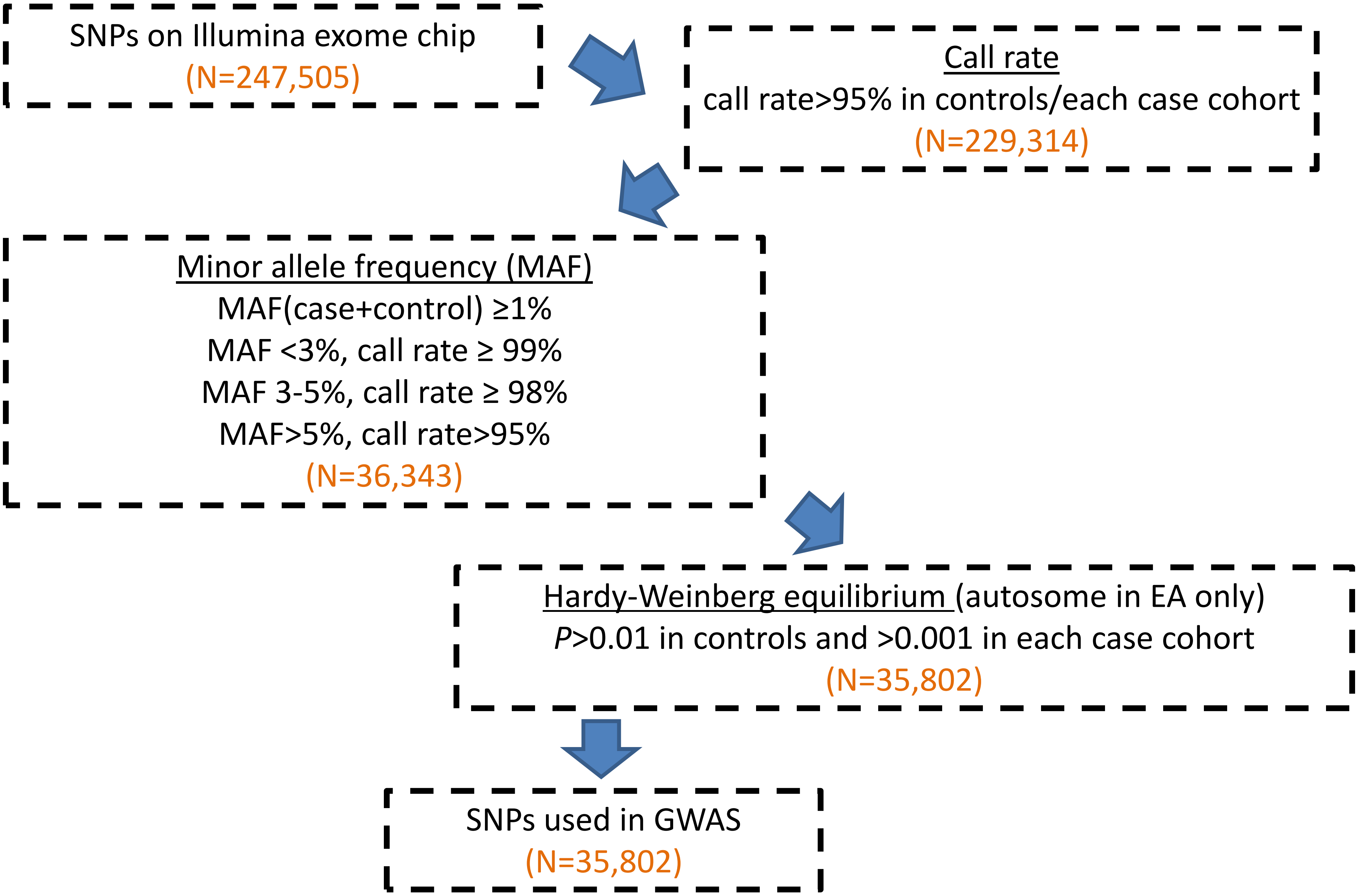


**Supplementary Figure 5. SNP quality control in the GWAS.** SNPs were filtered on the basis of allele frequency, call rate, and deviation from Hardy-Weinberg equilibrium, as detailed in Supplementary Text.

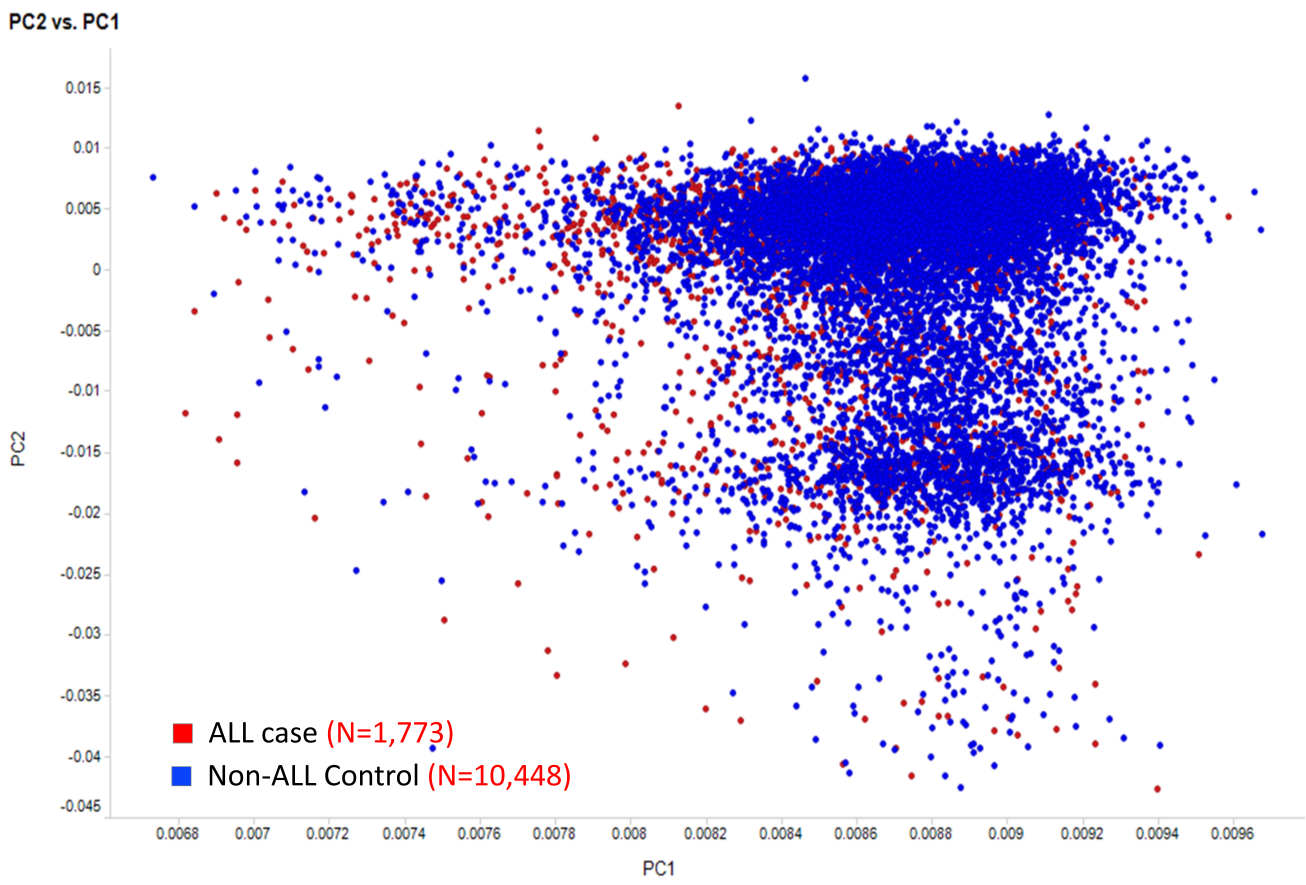


**Supplementary Figure 6. Principal components analysis (PCA) in the discovery GWAS series.** Principal components (PCs) were determined using EIGENSTRAT with genome-wide SNP genotype of both B-ALL cases and controls in the discovery GWAS series. Similar population structure in cases vs. controls is confirmed by the overlapping distribution of PC1 and PC2 of these two groups (N=1,773 and 10,448, respectively).

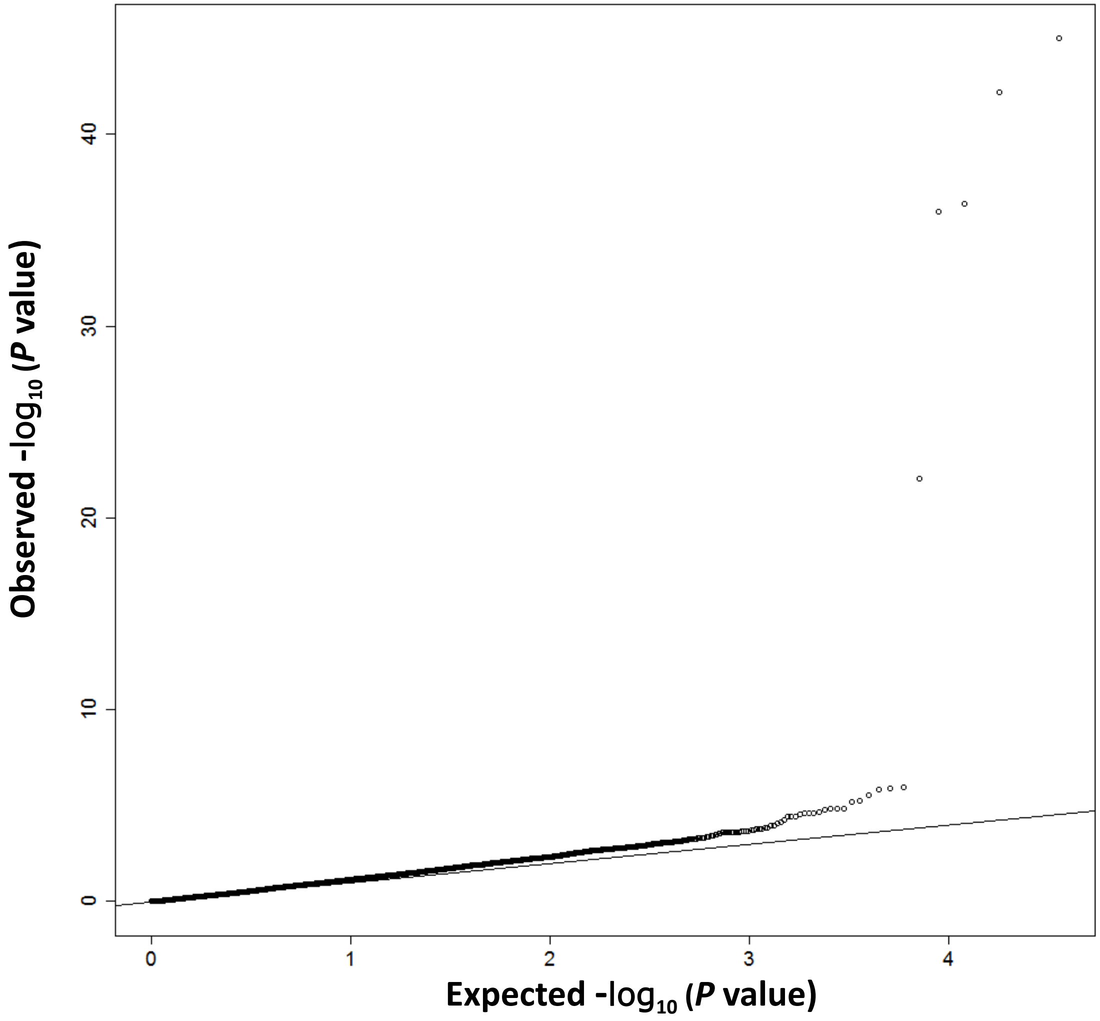


**Supplementary Figure 7. Quantile-quantile (Q-Q) plot of logistic regression test for GWAS.** The negative logarithm of the observed (y axis) and the expected (x axis) *P* value is plotted for each SNP (dot), and the black line indicates the null hypothesis of no true association. Deviation from the expected *P* value distribution is evident only in the tail area (λ=1.08), suggesting that population stratification was adequately controlled by adjusting for PCA.

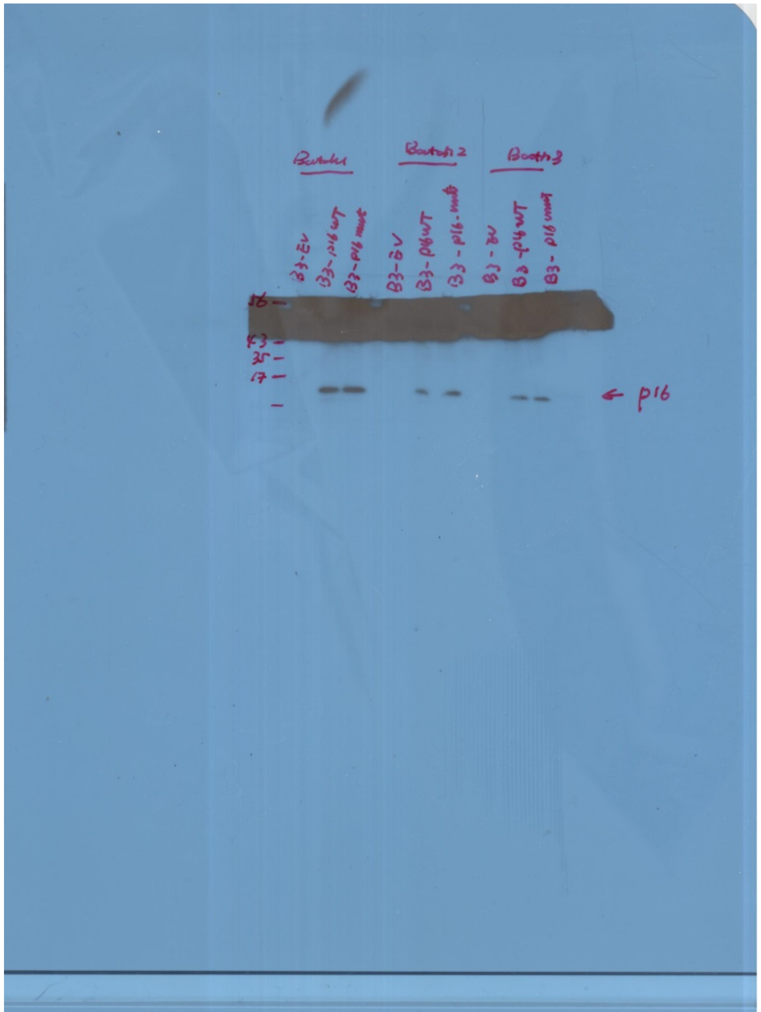


1 hour exposure for p16

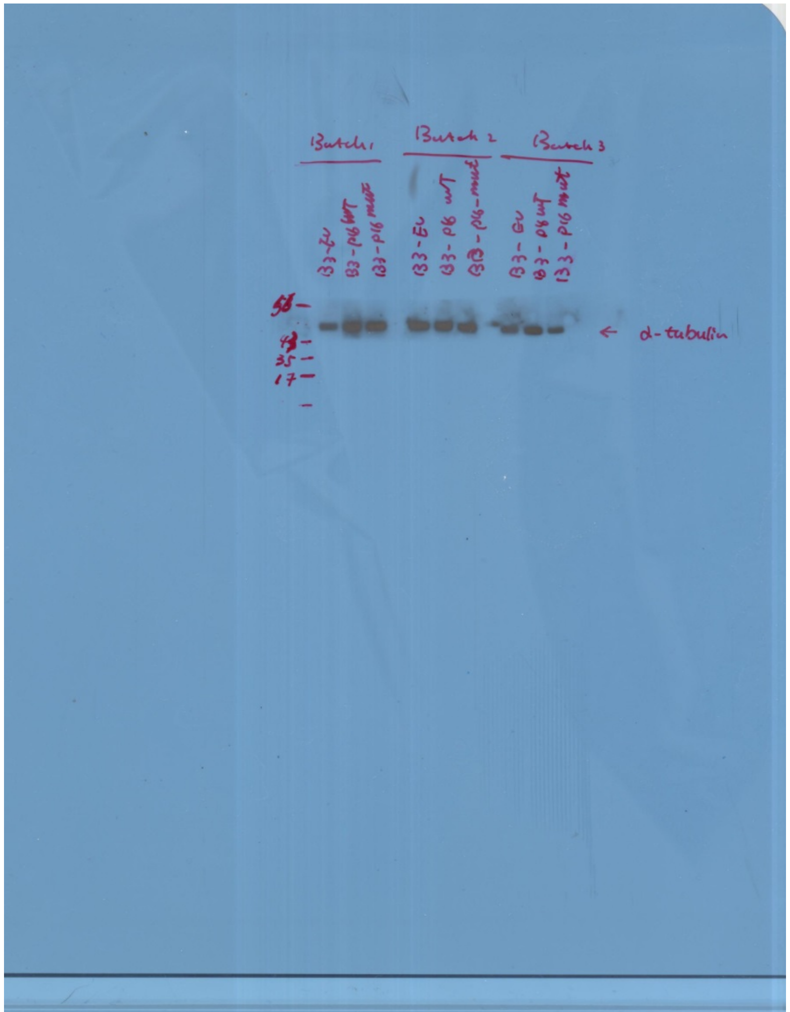


1 minute exposure for α-Tubulin

**Supplementary Figure 8. Uncropped scans of Western blot shown in Supplementary Figure 2a.**

**Supplementary Table 1. B-ALL susceptibility variants at the *ARID5B* and *IKZF1* loci in the discovery GWAS cohort**

| SNP | Chr | Position* | Gene | Alleles† | RAF (case/ctrl) | *P* value | OR‡ (95% CI) |
|---|---|---|---|---|---|---|---|
| rs10821936 | 10 | 63723577 | *ARID5B* | **C**/T | 0.462/0.331 | $9.9\times10^{-46}$ | 1.71 (1.59-1.84) |
| rs7089424 | 10 | 63752159 | *ARID5B* | **G**/T | 0.46/0.334 | $6\times10^{-43}$ | 1.68 (1.56-1.81) |
| rs4132601 | 7 | 50470604 | *IKZF1* | **G**/T | 0.378/0.268 | $4.3\times10^{-37}$ | 1.65 (1.52-1.78) |
| rs11978267 | 7 | 50466304 | *IKZF1* | A/**G** | 0.378/0.269 | $1.1\times10^{-36}$ | 1.64 (1.52-1.77) |

Abbreviations: Chr, chromosome; RAF, risk allele frequency; OR, odds ratio; CI, confidence interval
*Chromosomal locations are based on hg19; †Bold denotes the allele that had a significantly higher frequency in children with B-ALL than in the non-ALL controls (i.e., risk allele for B-ALL); ‡OR, odds ratio represents the increase in the risk of developing B-ALL for each copy of the risk allele compared with subjects who do not carry the risk allele; *P* values and ORs were estimated by the logistic regression test.

**Supplementary Table 2. Conditional analyses of independent associations at the *CDKN2A* locus**

| | | | univariate analyses | | multivariate analyses | |
|---|---|---|---|---|---|---|
| **SNP** | **Chr** | **Position*** | ***P* value†** | **OR‡ (95% CI)** | ***P* value** | **OR (95% CI)** |
| rs3731249 | 9 | 21970916 | $6\times10^{-22}$ | 2.23 (1.89-2.62) | $3.9\times10^{-20}$ | 2.15 (1.83-2.54) |
| rs3731217§ | 9 | 21984661 | $7.1\times10^{-9}$ | 1.42 (1.26-1.61) | $1.8\times10^{-7}$ | 1.38 (1.22-1.56) |

Abbreviations: Chr, chromosome; OR, odds ratio; CI, confidence interval
*Chromosomal locations are based on hg19; †*P* value was estimated by comparing 1,773 children with B-ALL and 9,590 unrelated non-ALL control from the ARIC cohort ; ‡OR, odds ratio represents the increase in the risk of developing B-ALL for each copy of the risk allele compared with subjects who do not carry the risk allele; §rs3731217 was the intronic variant in *CDKN2A* previously associated with B-ALL susceptibility (Nat Genet. 2010 Jun;42[6]:492-4); *P* values and ORs were estimated by the logistic regression test.

**Supplementary Table 3. *CDKN2A* and *CDKN2B* exonic germline variants identified in 2,407 childhood B-ALL cases by targeted resequencing**

| Gene | SNP ID | Genomic change (Chr. 9) | Nucleotide alteration | Amino acid change | Variant type | Allele frequency (%) | # Patients | Bioinformatic prediction tools (ref.23) CADD (scaled C score) |
|---|---|---|---|---|---|---|---|---|
| *CDKN2A:p16$^{INK4a}$* | - | g.21974720G>A | c.107C>T | p.Ala36Val | missense | 0.02 | 1 | 28.20 |
| | rs372266620 | g.21971188G>A | c.170C>T | p.Ala57Val | missense | 0.02 | 1 | 6.57 |
| | - | g.21971062C>T | c.296G>A | p.Arg99Glyln | missense | 0.02 | 1 | 10.66 |
| | rs199888003 | g.21971040C>T | c.318G>A | p.Val106Val | silent | 0.10 | 5 | 12.37 |
| | rs146179135 | g.21970985C>G | c.373G>C | p.Asp125His | missense | 0.04 | 2 | 12.25 |
| | rs6413464 | g.21970979C>A | c.379G>T | p.Ala127Ser | missense | 0.02 | 1 | 22.80 |
| | rs145012438 | g.21970946T>C | c.412A>G | p.Arg138Gly | missense | 0.02 | 1 | 8.97 |
| | - | g.21970933T>C | c.425A>G | p.His142Arg | missense | 0.02 | 1 | 10.80 |
| | - | g.21970931C>T | c.427G>A | p.Ala143Thr | missense | 0.02 | 1 | 4.44 |
| | rs3731249 | g.21970916C>T | c.442G>A | p.Ala148Thr | missense | 6.80 | 311 | 12.56 |
| *CDKN2A:p14$^{ARF}$* | - | g.21994324G>A | c.7C>T | p.Arg3Cys | missense | 0.02 | 1 | 24.90 |
| | - | g.21994308G>T | c.23C>A | p.Thr8Asn | missense | 0.02 | 1 | 18.95 |
| | rs374360796 | g.21994262G>A | c.69C>T | p.Phe23Phe | silent | 0.08 | 4 | 8.59 |
| | - | g.21994252T>G | c.79A>C | p.Ile27Leu | missense | 0.02 | 1 | 14.88 |
| | rs199888003 | g.21971040C>T | c.361G>A | p.Ala121Thr | missense | 0.10 | 5 | 12.37 |
| *CDKN2B:p15$^{INK4b}$* | - | g.22008918C>T | c.35G>A | p.Gly12Asp | missense | 0.02 | 1 | 18.04 |
| | rs375443156 | g.22008831T>C | c.122A>G | p.Asn41Ser | missense | 0.02 | 1 | 35.00 |
| | rs148421170 | g.22006147C>T | c.256G>A | p.Asp86Asn | missense | 0.27 | 13 | 33.00 |
| | - | g.22006108G>GCAC | c.294_295insGTG | p.Leu99ValLeu | in-frame insertion | 0.02 | 1 | - |
| | - | g.22006002G>A | c.401C>T | p.Thr134Ile | missense | 0.02 | 1 | 12.36 |
| | - | g.22005991C>A | c.412G>T | p.Asp138Tyr | missense | 0.02 | 1 | 13.48 |

**Supplementary Table 4. Plausible B-ALL susceptibility genes identified by gene-level SKAT test in the discovery GWAS cohort***

| Gene | Flanking Region | *P* value | Number of SNPs (included/all) | Chr | SNP Position | SNP ID† | case MAF (%) | control MAF (%) |
|---|---|---|---|---|---|---|---|---|
| *DYX1C1* | 55709953-55800432 | $1.7\times10^{-7}$ | 7/12 | 15 | 55664140 | rs199668969 | 0.056 | 0.034 |
| | | | | | 55664144 | rs35101262 | 0.056 | 0 |
| | | | | | 55731682 | rs116339967 | 0.79 | 0.201 |
| | | | | | 55759311 | rs201132774 | 0.113 | 0.034 |
| | | | | | 55789984 | rs114245686 | 0 | 0.01 |
| | | | | | 55790414 | rs16976354 | 0.056 | 0.014 |
| | | | | | 55790524 | rs143493699 | 0.169 | 0.148 |
| *ARRB1* | 74971165-75062875 | $1.3\times10^{-6}$ | 3/3 | 11 | 74978732 | rs140226575 | 0.085 | 0.005 |
| | | | | | 74985208 | rs78979036 | 0.113 | 0.014 |
| | | | | | 74988431 | rs142938698 | 0.028 | 0 |
| *CAPN5* | 76777991-76837198 | $1.8\times10^{-6}$ | 5/7 | 11 | 76795984 | rs149132399 | 0 | 0.005 |
| | | | | | 76804776 | rs144453005 | 0.282 | 0.029 |
| | | | | | 76825431 | rs202032027 | 0.028 | 0.038 |
| | | | | | 76834830 | rs114632044 | 0.028 | 0 |
| | | | | | 76834837 | rs143494790 | 0.056 | 0.01 |
| *FNIP2* | 159690181-159827954 | $4.6\times10^{-6}$ | 8/14 | 4 | 159747079 | rs62001910 | 0.056 | 0.014 |
| | | | | | 159780252 | rs148251675 | 3.36 | 2.48 |
| | | | | | 159780791 | rs199995620 | 0.113 | 0.034 |
| | | | | | 159782889 | rs62001915 | 2.62 | 2.14 |
| | | | | | 159789403 | rs147318337 | 1.47 | 1.1 |
| | | | | | 159789526 | rs188016133 | 0.028 | 0.024 |
| | | | | | 159789988 | rs199737425 | 0.028 | 0.005 |
| | | | | | 159812658 | rs200009550 | 0.056 | 0.019 |
| *GNAT1* | 50229042-50235129 | $9.9\times10^{-6}$ | 2/2 | 3 | 50229241 | rs149936603 | 0.028 | 0 |
| | | | | | 50231042 | rs141497735 | 0.113 | 0.005 |
| *FMNL3* | 50031723-50101197 | $1.5\times10^{-5}$ | 3/5 | 12 | 50042110 | rs199600439 | 0.028 | 0.005 |
| | | | | | 50047067 | rs116557867 | 0.028 | 0 |
| | | | | | 50100925 | rs200294583 | 0.199 | 0.024 |
| *CASP2* | 142985307-143004789 | $2.9\times10^{-5}$ | 2/2 | 7 | 142985603 | rs145693200 | 0.028 | 0 |
| | | | | | 142991379 | rs4647298 | 0.085 | 0 |

***only missense, stop-codon altering, and splice site variants with minor allele frequency <5% were included in the SKAT test. Association was estimated based on aggregated effects in children with B-ALL cases and non-ALL controls included in the discovery GWAS cohort; † Chromosomal locations are based on hg19.

**Supplementary Table 5. Missense SNPs nominally associated with B-ALL susceptibility (*P*<0.05) at the *CEBPE* locus**

| SNP ID | Chr | Position* | Gene | Alleles† | RAF% (case/ctrl) | Amino acid change | *P* value | OR‡ (95% CI) |
|---|---|---|---|---|---|---|---|---|
| rs141903485 | 14 | 23587838 | *CEBPE* | G/**T** | 0.29/0.93 | p.Leu155Met | $7.8\times10^{-4}$ | 0.33 (0.17-0.63) |
| rs146580935 | 14 | 23588096 | *CEBPE* | **A**/G | 0.14/0.06 | p.Leu69Phe | 0.05 | 2.81 (1-8.16) |

Abbreviations: Chr, chromosome; RAF, risk allele frequency; OR, odds ratio; CI, confidence interval
*Chromosomal locations are based on hg19; †Bold denotes minor allele at this SNP; ‡OR, odds ratio represents the increase in the risk of developing B-ALL for each copy of the risk allele compared with subjects who do not carry the minor allele; *P* values and ORs were estimated by the logistic regression test.
